# The impact of T cells on immune-related liver diseases: an overview

**DOI:** 10.1186/s41232-025-00387-0

**Published:** 2025-07-04

**Authors:** Yuzo Koda, Ryosuke Kasuga, Nobuhito Taniki, Takanori Kanai, Nobuhiro Nakamoto

**Affiliations:** 1 Division of Gastroenterology and Hepatology, Department of Internal Medicine, Keio University School of Medicine, Tokyo, Japan; 2 Mitsubishi Tanabe Pharma Corporation, Kanagawa, Japan; 3 Japan Agency for Medical Research and Development, AMED, Tokyo, Japan

**Keywords:** T cells, CD4^+^ T cells, CD8^+^ T cells, Resident memory T cells, Immune-related liver diseases

## Abstract

The liver presents a unique immune system. Liver diseases are closely associated with the immune system. Disruption of the tightly regulated balance between immune activation and tolerance induction leads to the development and worsening of immune-related liver diseases. T cells play diverse crucial roles in the immune system, and they have long been known to induce inflammation through direct tissue damage by effector molecules and the recruitment of effector cells via chemokines. Additionally, T cells interact with B cells to induce autoantibodies, promoting tissue inflammation and dysfunction through the deposition of IgG and immune complexes in the tissues. Recent advances in omics technologies, including single-cell RNA sequencing and spatial transcriptomics, have elucidated the role of T cells in the progression and recovery of liver fibrosis. Moreover, comprehensive and unbiased information can now be obtained from small samples of human and mouse tissues, which advances our understanding of tissue-specific functions of T cells, including resident memory T cells, peripheral helper T cells, and tissue Tregs. However, significant unmet needs remain in the fields of immune-related liver diseases. In this review, we discuss the T cell biology and its role in autoimmune hepatitis (AIH), primary sclerosing cholangitis (PSC), primary biliary cholangitis (PBC), and metabolic-associated steatohepatitis (MASH), which are non-viral liver diseases exhibiting a strong involvement of immunity and inflammation. Furthermore, the latest therapeutic concepts for the diseases and associated drugs targeting T cells have been overviewed.

## Introduction

The liver is exposed to pathogens and antigens from the intestine [1]. T cells are essential to respond appropriately to various antigens to which they are exposed and to induce an effective immune response. They regulate responses to pathogens and eliminate them via antigen-presenting cells; moreover, induction of tolerance to prevent excessive immune responses is regulated by T cells in the steady state [1]. The liver is a unique organ in which interactions between circulating blood T cells and liver antigen-presenting cells occur within the sinusoids, resulting in a distinctive T cell regulatory mechanism [1]. Moreover, the liver has a specific immune microenvironment associated with factors such as CXCL16 produced by liver sinusoidal endothelial cells (LSECs) [2]. The unique structure comprising hepatic sinusoids, Kupffer cells, endothelial cells, and stellate cells gives different roles based on the position of immune cells [3]. In addition to conventional CD8^+^ T cells and CD4^+^ T cells, innate-like T cells such as natural killer T (NKT), γδ T, and mucosal-associated invariant T (MAIT) cells are more abundant in the liver than in other organs [4]. The abundance of these cells in the liver is considered necessary for the effective processing of incoming antigens, recognition of a narrow range of antigens, including lipids, glycolipids, and antigens of bacterial origin, and the rapid and massive release of cytokines [5]. In immune-related liver diseases, these unique immune cell populations interact in complex ways, contributing to disease progression and exacerbation. In particular, T cells play a significant role in initiating the activation of the innate immune system, including macrophages and neutrophils, and induce autoantibody production through cytokine and chemokine synthesis. However, accumulating evidence demonstrates that during the improvement of liver fibrosis, these CD8^+^ T cells attack fibrogenic cells such as hepatic stellate cells (HSCs) and contribute to the improvement of the disease state [4, 6]. Chronic liver disease progresses through the initial stages of acute inflammation, chronic inflammation, fibrosis, and cirrhosis; the role of T cells varies at each stage [4]. As the understanding of metabolic dysfunction-associated steatohepatitis (MASH) pathology shifts from the two-hit theory to the multiple-hit theory, the pathology of each phase progresses in a gradient, increasing the complexity of understanding the pathology and T cell biology.

In this review, we focus on four diseases: immune-related liver diseases including autoimmune hepatitis (AIH), primary biliary cholangitis (PBC), primary sclerosing cholangitis (PSC), and MASH, which involve immune cells, and provide an overview of the roles and functions of T cells in each liver disease.

### Differences in the roles of T cells in immune-related liver diseases

**Fig. 1:**
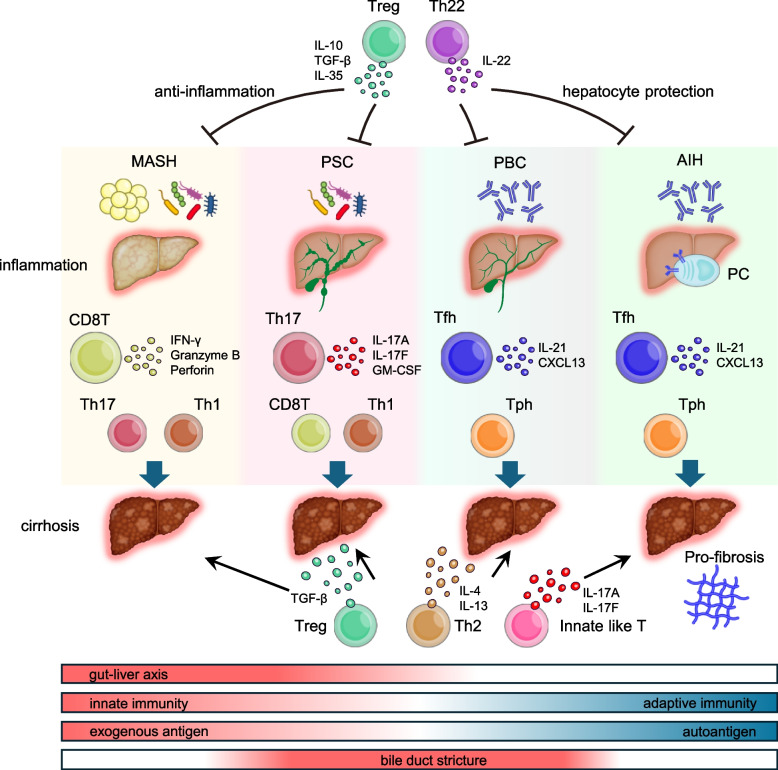
Differences in the roles of T cells in immune-related liver diseases An overview of the correlation between each liver disease and T cell subsets. *Treg* regulatory T cells, *Th* T helper, *Tfh* follicular helper T, *Tph* peripheral helper T, *PC* plasma cells, *MASH* metabolic dysfunction-associated steatohepatitis, *PSC* primary sclerosing cholangitis, *PBC* primary biliary cholangitis, *AIH* autoimmune hepatitis, *TGF* tumor growth factor, *IL* interleukin

T cell subsets in the liver can be broadly classified into CD8^+^ T cells, CD4^+^ T cells, and innate-like T cells (γδ T cells, NKT cells, MAIT cells). CD4^+^ T cells are further classified into various subsets, including Th1 cells, which induce cellular immunity and activate CD8^+^ T cells, Th2 cells that induce allergic responses, Th17 cells that produce IL-17A and recruit neutrophils, and Tregs, which suppress excessive immune cell activation [7]. Follicular helper T (Tfh) cells help B cells mature and induce antibody production in lymphoid tissues [8, 9]. Recently, a new subset of peripheral helper T (Tph) cells was identified, which form tertiary lymphoid organs (TLOs) and induce antibody production at sites of inflammation rather than in lymphoid tissues [10]. Although the complete pathological picture of immune-related liver diseases remains unclear, each disease is characterized by specific immunological characteristics and abundance in different cell populations. An overview of this process is shown in Fig. 1.

CD8^+^ T cells and Th1/Th17 cells, which promote inflammation and cellular immunity, are significantly involved in MASH and PSC among immune-related liver diseases [11, 12]. However, the contribution of Tfh/Tph and B cells, which promote adaptive immunity, to the pathology of these diseases has not been widely reported. MASH and PSC frequently lead to leaky gut owing to an abnormal composition of the gut microbiota and increased intestinal permeability, exposing the liver to bacteria and other external antigens, which likely leads to the induction of both innate and adaptive immune systems [13–15]. In MASH, the skew toward CD8^+^ T cells is particularly notable, and the role of CD8^+^ T cells in pathology is gradually being elucidated [16]. However, the detailed mechanisms underlying the increased abundance of CD8^+^ T cells in MASH remain unclear. In PSC, the skew toward Th17 cells is well known, and bacteria that induce liver Th17 cells have been identified [17]. Recently, the accumulation of CD8^+^ tissue-resident memory T cells (Trm) has been reported in the bile ducts of patients with PSC [18].

However, the contribution of cells involved in adaptive immunity, such as Tfh and B cells, in AIH and PBC has been strongly suggested. In AIH, autoantibodies such as antinuclear, anti-smooth muscle, and liver-kidney microsomal antibodies have been identified, whereas, in PBC, anti-mitochondrial antibodies were identified as autoantibodies associated with the disease [19]. The effectiveness of B cell depletion therapies such as rituximab in corticosteroid- or ursodeoxycholic acid (UDCA)-resistant cases further suggests a stronger involvement of the adaptive immune system in these diseases [20]. In AIH and PBC, the contribution of external antigens is less than that in MASH and PSC, and reports suggesting the involvement of innate immune-like T cells are rare (Tfh and Tph cells section). However, several studies have indicated that Th1 and Th17 cells contribute to both diseases (Th1 and Th17 cells section). Moreover, the contributions of CD8^+^ T and Th22 cells have also been reported in AIH, indicating the involvement of a relatively wide range of T cells (CD8^+^ T and Th22 cells section). Treg cells have been reported to have immunosuppressive and pro-fibrotic effects across immune-related liver diseases (Treg section). Th2 cells are suggested to be involved in fibrosis, a common progressive pathology in all these diseases, through IL-4- and IL-13-mediated induction, but specific reports are scarce, and the details remain unclear (Th2 cells section). The following sections present a detailed overview of each T cell subset and their involvement in liver diseases.

### *CD8*^+^*T cells*

CD8^+^ T cells, historically known as cytotoxic T lymphocytes (CTLs), play an important role in immune defense against intracellular pathogens, including viruses and bacteria, and in antitumor immunity [21]. Similar to CD4^+^ T cell subsets, the concept of CD8^+^ T cell subsets, such as Tc1, Tc2, Tc9, Tc17, and CD8^+^ Tregs, has recently been proposed [21, 22]. However, the association of these subsets with immune-related liver diseases has not been well analyzed. Therefore, in this section, we outline the role of classical CD8^+^ T cells, classified as Tc1 cells, which are commonly referred to as CTLs. CD8^+^ T cells are activated under IL-2/IL-12 stimulation and exert potent cytotoxic activity by producing effector molecules such as interferon gamma (IFN-γ), granzyme, and perforin [21]. They also induce apoptosis in target cells through FasL-mediated direct cell–cell interactions [21].

Activation of CD8^+^ T cells and their cytotoxicity against hepatocytes were reported in immune-related liver diseases, such as MASH, AIH, and PSC, primarily in mouse models. Recent studies have elucidated the detrimental role of CD8^+^ T cells in MASH-related inflammation and fibrosis [23–25]. In 2004, Safadi et al. reported that the transfer of CD8^+^ T cells into severe combined immune-deficient (SCID) mice induced fibrosis by carbon tetrachloride (CCl4) and thioacetamide (TAA), while the transfer of CD4^+^ T cells did not [26]. A study reported in Cancer Cell in 2014 showed an increased abundance of intrahepatic CD8^+^ T and NKT cells in a mouse model of choline-deficient high-fat diet (CDHFD)-induced MASH; the phenotype was canceled in RAG1 knock out (KO) mice (lacking T cells, B cells, and NKT cells) and β2mKO mice (with non-functional CD8^+^ T cells and NKT cells), suggesting the involvement of CD8^+^ T cells in MASH [23]. In 2017, it was demonstrated that a high-fat, high-carbohydrate diet induced MASH pathology and increased the number of CD8^+^ T cells in the liver [24]. The depletion of CD8^+^ T cells using anti-CD8 antibodies reduced the level of liver inflammation markers (ALT), macrophage infiltration, and fibrosis markers (αSMA, col1a1), directly indicating the detrimental role of CD8^+^ T cells in MASH. In 2021, a detailed characterization of CD8^+^ T cells and the mechanisms by which they exacerbate MASH pathogenesis were proposed; CXCR6-expressing autoreactive CD8^+^ T cells were increased in both the mouse model of MASH (CDHFD). Interestingly, these cells exacerbate MASH by damaging hepatocytes independently of major histocompatibility complex (MHC) class I [25]. The pathogenic role of CD8^+^ T cells in MASH patients is suggested to be associated with a mechanism similar to that detected in mice.

The importance of CD8^+^ T cells in the pathogenesis of PSC has been demonstrated using MDR2KO mice, a PSC model. Depletion of CD8^+^ T cells using anti-CD8 antibodies controlled osteopontin production in the liver and improved bile duct fibrosis caused by MDR2 deficiency [27]. As osteopontin production is upregulated in bile duct epithelial cells exposed to injury or cytokine stimulation [28], it is assumed that the depletion of CD8^+^ T cells reduced direct cell damage to the bile ducts and suppressed pro-fibrogenic osteopontin. A previous report revealed that depletion of lymphocytes, including CD8^+^ T cells, with anti-Thy1 antibody (Ab) or in RAG1KO, improved cholangitis in MDR2KO mice via suppressed IFN-γ expression [29]. The splenic CD8^+^ T cells induced by IL-15-producing B cells contribute to the AIH pathology in mice [30]. Moreover, CD8^+^ T cells contribute to the pathology of general fibrosis, regardless of the underlying disease [12]. When SCID mice with liver fibrosis induced by CCl4 or TAA were transferred with CD8^+^ T cells or CD4^+^ T cells, liver fibrosis was exacerbated through CD8^+^ T cell transfer, reflecting the contribution of CD8^+^ T cells to the pathogenesis of fibrosis [26]. Furthermore, CD8^+^ T cells regulate macrophages in a perforin-dependent manner and suppress the pathogenesis of MCD in a mouse model of MASH, indicating that CD8^+^ T cells potentially act against disease-exacerbating cells and protect against MASH pathology [31].

While exacerbating the pathological condition, CD8^+^ T cells tend to interact with hepatocytes, whereas they interact with HSCs or other cells to function protectively [6, 31]. The differences between the fibrotic exacerbation and recovery phases associated with CD8^+^ T cells potentially depend on the ease of cell–cell interactions in each phase. Our study suggests that interactions between CD8^+^ T cells and hepatocytes likely occur via the CCR5 axis during the fibrotic exacerbation phase, and CD8^+^ T cells interact with HSCs during the recovery phase [6]. These differences potentially contribute to the varying roles of CD8^+^ T cells. Additionally, as cell–cell interactions are intricately regulated, the primary cell–cell interactions may differ depending on the conditions inducing the pathology. Furthermore, single-cell RNA sequencing revealed differential gene expression profiles in CD8^+^ T cells between the exacerbation and recovery phases [6], indicating that changes in the characteristics of CD8^+^ T cells may alter their roles. However, their physiological and pathological roles need to be further elucidated. Trm, a recently identified cell subset, is considered an important perspective for understanding CD8^+^ T cells. The following section provides an overview of Trm cells and their significance in immune-related liver diseases.

### Tissue resident memory T cells

Recently, the concept of Trm has been introduced, which refers to lymphocytes present and maintained in tissues, exhibiting no circulation through the blood or lymphatic system [32–34]. Trm cells exist in the liver under both normal and pathological conditions. They are characterized by the expression of transcription factors including B lymphocyte-induced maturation protein 1 (BLIMP1), HOBIT, TOX, and EOMES [33]. In the liver, IL-15 and/or TGF-β1 have been identified as important factors for survival and differentiation within tissues [6, 33, 35, 36]. Trm cells are primarily marked by surface antigens such as CD69, CD103, CD49a, CXCR6, and CXCR3, crucial for anchoring them to tissues [12]. Conversely, S1P1, CCR7, and KLF2 are downregulated in these cells [12]. The pathology-related role of Trm cells has been reported mainly in viral liver diseases [35, 37, 38]. However, CD8^+^ T cells exhibit a memory phenotype and contribute to the pathogenesis of immune-related liver diseases such as MASH, PSC, and AIH [6, 18, 30]. Our group demonstrated that CD69^+^CD103^−^CXCR6^+^CD8^+^ Trm cells contribute to disease recovery in a mouse model of MASH induced by a high-fat, high-carbohydrate diet through apoptosis in HSCs via FasL during the fibrosis recovery [6]. Further investigations can validate whether similar mechanisms occur in pathologies other than MASH. Moreover, an increased abundance of CD8^+^ Trm cells is reported in human MASH pathology [6]. Unlike the prevalence of CD103-negative CD8^+^ Trm cells in the liver of mice, a CD103-positive population has been reported in humans [18, 39], suggesting a potential difference in the mechanism, including differences in E-cadherin expression patterns, between mice and humans.

Reports on Trm cells associated with immune-related liver diseases in mice are limited. A study demonstrated that Trm-like cells that cause bile duct injury are increased in an IL-12β/IL2-Rα double KO-induced PBC mouse model, and their removal improves the pathology [40]. However, analysis of Trm cells is progressing in immune-related diseases in humans. The number of CD69^+^CD103^+^CD8^+^ Trm-like cells increases in the liver and is correlated with corticosteroid responsiveness in patients with AIH [41]. The mechanism of Blimp1-mediated induction of Trm by TGF-β1 and IL-15 has been previously demonstrated [41]. CD103^+^CD8^+^ T cells infiltrate the liver in patients with acute liver failure with an AIH background, contributing to the pathology [41]. Multiparameter fluorescence-activated cell sorting (FACS) analysis has revealed that the most common resident T cells infiltrating the peribiliary tract in human patients with PSC are CD103^+^CD69^+^ CD8^+^ T cells, which are considered T effector memory cells; however, this cell population expressing the core gene signature of Trm, including CXCR6, overlaps with the Trm population [18]. These CD8^+^ T cells express high levels of CXCL8 and IL-17 in the liver, induce the accumulation of neutrophils, and are involved in organ damage and the induction of innate immune responses. Interestingly, these cells also express gut-homing receptors such as α4β7 and CCR9 [42]. The concept reflecting that CCR9-positive T cells are key to the gut-liver axis has long been proposed in humans [43]; recently, detailed next-generation analyses of local human cells have started revealing the specificity of these cells [44, 45]. Spatial transcriptomics of the human fibrotic liver revealed significantly higher frequencies of specific cell subsets, including HSCs, monocytes, Kupffer cells, T cells, and B cells, in fibrotic liver regions compared to those in parenchymal areas of cirrhotic explants [44]. Another report presenting the spatial transcriptome of the human small intestine reveals the diversity of CD8^+^ Trm and elucidates the mechanism underlying their accumulation, which is mediated by the CXCR3 axis [45].

However, the specific roles of Trm in immune-related liver diseases require further elucidation. In addition, most Trm analyses reported in the liver are for CD8^+^ T cells, indicating that further studies are needed on the characteristics and properties of CD4^+^ Trm.

### Mechanisms underlying tissue residency of liver Trm cells

Homing of T cells to the liver under steady-state conditions is controlled by adhesion molecules and chemokine receptors such as ICAM1 and CXCR6 [46–48]. In inflammatory or pathological conditions, it is regulated by inflammation-related chemokine receptors such as CCR2 and CXCR3, and gut-homing receptors, such as CCR9 and α4β7, which are associated with the gut-liver axis [42, 43, 46, 48, 49]. Recent studies have proposed several mechanisms associated with the tissue residency of Trm cells.

CXCR6 expressed on Trm cells senses CXCL16 produced by liver sinusoidal endothelial cells (LSECs) and attracts these cells to the perisinusoidal area [47, 50]. CXCR6 is also expressed in other liver-specific tissue-resident cells, such as NKT cells, contributing to the unique immune microenvironment of the liver at a steady state [2].

**Fig. 2:**
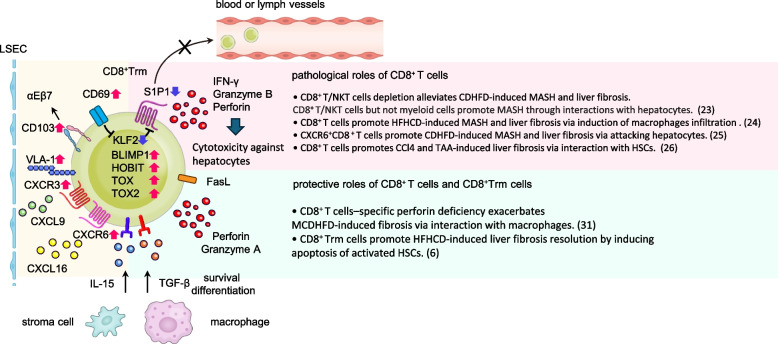
The roles in immune-related liver diseases and tissue residency mechanisms of liver Trm cells An overview of the role of Trm cells and associated tissue residency-related mechanisms. CD8^+^ Trm cells exhibit upregulation of transcription factors such as BLIMP1, HOBIT, TOX, TOX2 [33]. Conversely, increased expression of CD69 and subsequent downregulation of KLF2 and S1P1, which promotes circulation of lymphocytes in the blood, induces tissue retention [12, 51, 52]. Direct binding to tissues via adhesion molecules such as CD103 and VLA-1 and chemotaxis to tissues via CXCR6 and CXCR3 are also thought to contribute to tissue residency of Trm cells [12]. The survival is maintained by cytokines such as IL-15 and TGF-β1 produced by macrophages and stroma cells [6, 33, 35, 36]. The pathogenic role of CD8^+^ T cells in liver pathology involves primarily their direct action on hepatocytes [23, 25]. The involvement on macrophages are controversial [23, 24], and there are reports that CD8^+^ T cells promote fibrosis through their interaction with HSCs [26]. As a protective role in liver pathology, induction of macrophage polarization via perforin produced by CD8^+^ T cells has been reported. Our group has shown that CD8^+^ Trm cells promote the resolution of liver fibrosis by inducing apoptosis of activated HSCs[6]. *Trm* tissue resident memory, *LSEC* liver sinusoidal endothelial cell, *CDHFD* choline-deficient high-fat diet, *HFHCD* high-fat high-carbohydrate or cholesterol diet, *MCDHFD* methionine choline-deficient high-fat diet, *CCl4* carbon tetrachloride, *TAA* thioacetamide

CD69, presenting a Trm marker and lectin, is induced by the suppression of KLF2 during T cell activation. CD69 binds to S1P1, which is important for its migration into the bloodstream and lymphatics, and induces its internalization and degradation; hence, Trm cells are retained in the tissue [51, 52]. Moreover, Trm marker CD103 is the α chain of integrin αEβ7 and is induced by TGF-β [53]. Its ligand, E-cadherin, is highly expressed in hepatocytes and cholangiocytes, suggesting an important role of CD103 in tissue residency of Trm cells [18, 54]. CD49a is a component of the integrin VLA1 (α1β1) and binds to collagen IV [55]. Additionally, LFA-1, a molecule involved in liver homing, was recently identified and considered a crucial factor for the retention of Trm cells in the liver [56]. An overview of the role and tissue residency-related mechanisms of Trm cells is depicted in Fig. 2.

Although multiple mechanisms for maintaining Trm cells in tissues have been elucidated, the mechanisms of tissue differentiation and plasticity of Trm cells compared to other CD8^+^ T cell types, such as effector memory cells and central memory cells, remain unclear.

### Th1 cells

Th1 cells, comprising a subset of CD4^+^ T cells, are regulated by the master transcription factor T-bet [7, 57]. Under normal conditions, they contribute to the defense against intracellular viral and bacterial infections via effector molecules, including IFN-γ and activated macrophages [7, 57]. Antigen-stimulated T cells, along with IL-12 and IFN-γ signaling, differentiate into Th1 cells via STAT1/4 [58]. Several previous reports demonstrate an increased number of Th1 cells in the blood or liver in immune-related liver diseases and associated macrophage activation, suggesting a positive correlation of Th1 cells with pathogenesis.

Several studies demonstrated the involvement of IFN-γ-producing Th1 cells induced by Concanavalin A (ConA) in mouse models of hepatitis [59, 60]. However, the contribution of these cells to the pathology of each mouse model of immune-related hepatitis needs further clarification. Conversely, reports on variations in Th1 cells in human patients have been elucidated across various immune-related hepatitis cases. In patients with AIH, the blood levels of Th1 cells show a strong correlation with disease progression, and IFN-γ-producing T cells are elevated in the liver [61]. IFN-γ-positive Th1 cells, as well as Th17 and CD8^+^ T cells, are predominant in the bile ducts of human patients with PSC [18]; however, their exact role remains unknown. In the MASH, analyses of patients with metabolic dysfunction-associated steatotic liver disease (MASLD) have reported an increase in Th1 cells in the blood [62]. Although several studies have documented the involvement of Th1 cells in obesity-induced adipose inflammation, their involvement in liver-specific inflammation and fibrosis in MASH has rarely been reported. The contribution of CD8^+^ T and Th17 cells in the local liver pathology of MASH is strongly suggested [16, 62–65]. Direct illustration of the involvement of Th1 cells in PBC remains limited. However, GWAS analyses have identified Th1-inducing factors such as IL12A, IL12B, IL12RB2, and STAT4 as susceptibility variants [66]. However, the lack of changes in Th1 cells or IFN-γ in the blood of patients with PBC and the ineffectiveness of ustekinumab, which neutralizes IL-12/23 p40, as reflected in clinical trials for PBC, suggest that Th1 cells may contribute a little to the pathology [67]. With respect to fibrosis, Th1 cells may play an antifibrotic role by balancing fibrosis-promoting type II-related cells, such as Th2 cells and ILC2s [68]. This potential role should be further examined in immune-related liver diseases.

### Th2 and Th9 cells

Th2 and Th9 cells comprise a subset of CD4^+^ T cells. Th2 cells are regulated by the master transcription factor GATA-3 [7, 57]. Under normal conditions, they contribute to the defense against extracellular parasites, bacteria, and toxins through the production of cytokines such as IL-4, IL-5, and IL-13; antibody production; and the activation of eosinophils and basophils. T cells that receive type II cytokine signals, such as those involving IL-4/IL-5 or IL-33/IL-25/IL-33/TSLP, differentiate into Th2 cells via STAT6/STAT5 [57]. Th2 cells are involved in fibrotic pathologies as well as allergic and autoimmune diseases [68–70]. Compared with Th1/Th17 cells, Th2 cells are less explored in the context of their involvement in the pathogenesis of immune-related liver diseases. In mouse models, IL-33 exacerbates overall liver fibrosis pathology in TAA or CCl4-induced hepatitis, suggesting that Th2 cells promote fibrosis [69]. In a high-fat diet-induced MASH model, Th2 cell cytokines promote fibrosis in mice deficient in Th1/Th2-related factors such as IL-4, IFN-γ, and IL-10 [71]. Furthermore, in a mouse model of bleomycin-induced pulmonary fibrosis, type 2 cytokines such as IL-13 and IL-33 were reported to promote disease exacerbation, supporting the role of Th2 cells in promoting hepatic fibrosis [70, 72]. IL-13 directly stimulates fibroblasts to produce collagen [73, 74], indicating that Th2 cells directly stimulate stellate cells in the liver to promote fibrosis. However, sources of type II cytokines are not limited to Th2 cells; eosinophils, mast cells, and ILCs also generate these cytokines [68]. The contribution of Th2 cells is possibly insignificant in immune-related human liver diseases because a strong induction of Th2 cells is rarely observed. Future studies should focus on the cell-specific involvement of type II cytokines in the pathogenesis of immune-related liver diseases and clarify the contribution of Th2 cells to fibrotic pathology.

Th9 cells produce IL-9 and induce a type 2 immune response similar to that of Th2 cells; they play important roles in parasitic infections [7]. Th9 cells are induced by co-stimulation with IL-4 and TGF-β, with PU.1 and IRF4 functioning as differentiation-related transcription factors. The contribution of Th9 cells to immune-related liver diseases remains unclear and requires further analysis; however, IL-9 levels in patients’ blood are reported to be associated with liver fibrosis and cirrhosis [75].

### Tfh and Tph cells

Tfh cells, comprising another subset of CD4^+^ T cells, are regulated by the master transcription factor B cell lymphoma 6 (BCL6) [76–78]. They were identified as T cells that help B cell maturation in the germinal center. Tfh cells are defined as PD-1^+^CXCR5^+^CD4^+^ T cells and are characterized by the expression of the chemokine receptor CXCR5, which is required for migration to the B cell region within lymph follicles [79]. IL-12/TGF-β signals induce human Tfh cells, which interact with B cells through the production of IL-21 and co-stimulatory molecules such as CD40L to promote B cell maturation and antibody production [80–83].

The Tfh cell differentiation differs between humans and mice [8], and few reports demonstrate the contribution of Tfh cells in immune-related hepatitis in mouse models. However, in human patients, a significant contribution of autoantibodies in AIH and PBC is evident. In AIH, the infiltration of plasma cells (PCs) in the liver tissue is essential for diagnosis; moreover, its correlations with liver-specific antigens, including anti-nuclear, anti-smooth muscle, anti-liver-kidney microsomal type 1 or 3 (anti-LKM-1, anti-LKM-3), anti-liver cytosol type 1 (anti-LC1), and anti-soluble liver (anti-SLA) antigens, have been identified [84, 85]. Tfh-related factors such as circulating Tfh cells, IL-21, and plasma cells correlate with IgG levels and disease activity in patients with AIH; additionally, T cell markers, such as programmed cell death protein 1 (PD-1) and CD38, are correlated with disease activity [86, 87]. Several studies have reported the contribution of Tfh cells in patients with PBC [20, 88]. The abundance of Tfh cells is increased in the liver and blood of patients with PBC, and co-culturing these cells with B cells enhances the production of antimitochondrial antibodies (AMAs) [20]. IL-21 also increases in the blood of treatment-naïve patients with PBC and decreases with disease improvement after UDCA treatment [20]. Genetic analyses have shown that risk loci in patients with PBC are located in genes encoding Tfh cell-associated proteins such as CXCR5 and CD40L [89]; hence, abnormal T-B interactions mediated by Tfh cells potentially contribute to the pathogenesis of the disease.

Contrastingly, the involvement of autoantibodies based on T-B interactions is potentially lower in PSC and MASH than in AIH and PBC [19]. Consistent with the involvement of the Th17 cell/neutrophil axis, autoantibodies against neutrophil-derived antigens (ANCA) are induced [90] and autoantibodies against gut-associated antigens (GP2) have been reported in patients with PSC-related complicated inflammatory bowel disease (IBD) [91, 92]. However, the association between these diseases and their contribution to them remains unclear. A recent report has suggested a direct link between Tfh cells and liver pathology in MASH [93]; however, IgA production and Tfh cells in Peyer’s patches of the gut regulate liver inflammation through gut homeostasis [93], suggesting their indirect contribution via the gut.

Recently, synovial tissue analysis of patients with rheumatoid arthritis identified Tph cells as a T cell subset that interacts with B cells and induces antibody production and ectopic lymphoid follicle formation in non-lymphoid tissues [10]. Similar to Tfh cells, Tph cells induce autoantibody production through the generation of IL-21 and CXCL13 in non-lymphoid tissues; however, they are defined as PD-1^+^CXCR5^−^CD4^+^ T cells lacking CXCR5 expression [9]. Tph cells are recruited to pathological tissues via inflammation-related chemokine receptors, such as CCR2, CCR5, CCR9, CX3CR1, and CXCR3. BCL6 and MAF are key transcription factors regulating Tfh cell differentiation; moreover, Tph cells are influenced by transcription factors such as BLIMP1, MAF, and SOX4 [9, 94–96]; however, their full characterization needs further validation.

The concept of Tph cells has been established through patient study, and the definition of Tph cells in rodents remains unclear. Hence, knowledge of Tph cells in rodents is mainly based on human-based analyses. Tph cells are involved in the pathogenesis of AIH and PBC, in which autoantibodies are strongly involved. In AIH, most auto-reactive CD4^+^ T cells specific to the soluble liver antigen (SLA) antigen exhibit Tph-like characteristics and promote B cell differentiation via IL-21 [97]. In patients with AIH, no significant increase in the activated Tfh cell population was reported in the blood, whereas the activated Tph cell population was considerably increased, which was strongly correlated with activated CD8^+^ T cells in the blood. In PBC, the percentage of Tph cells in the blood increases, and Tph cells expressing ICOS correlate with anti-mitochondrial antibodies and the number of plasma cells; their proportions were significantly reduced by UDCA treatment, suggesting their strong involvement in the pathogenesis [49].

Consistent with the low significance of B cell activation and autoantibodies in MASH and PSC, reports on Tph cells are scant and indicate a possible low contribution; however, further confirmation is needed. Tph cells have been reported in humans but not in rodents. Therefore, analysis of human samples is needed to validate their functions and roles. Analyzing the involvement of Tfh/Tph in human pathology using mouse models is challenging, especially because CXCL13, exhibiting important functional and dynamic roles in the associated processes, is differently distributed in mice [98]. To address this issue, an in vivo system that reflects human Tfh/Tph cells, such as a humanized immune system in mice, can be constructed.

### Treg cells

Treg cells belonging to another subset of CD4 T cells are regulated by the master transcription factor FOXP3 [99]. They play a crucial role in suppressing immune responses, maintaining immune tolerance, and maintaining immune cell homeostasis [99, 100]. Treg cells exert their immunosuppressive function through various mechanisms, including cell-contact mechanisms involving CTLA-4 and T cell immunoreceptor with Ig and ITIM domains (TIGIT), production of immunoregulatory cytokines such as IL-10, TGF-β1, and IL-35, and induction of apoptosis in target cells via granzymes [101]. The absence of Treg cells in a steady state causes systemic inflammation, as detected in the IPEX syndrome [102]. In disease states, Treg cells are known to play a crucial role in the regulation of immune responses in SLE, T1D, and rheumatoid arthritis (RA) [99, 103].

Treg cells are classified into two types: Helios-positive thymic Tregs, which are induced in the thymus, and retinoic acid-related orphan receptor gamma t (RORγt)-positive peripheral Tregs, which are induced in peripheral tissues such as the intestine [100]. Thymic Tregs are predominantly present in the liver and are increased in response to the induction of immune-related liver diseases [104]. Treg cells are often analyzed as a counterpart of the Th17 cell population, and their behavior has been reported to be altered in PSC, MASH, and AIH, indicating their significant role in these diseases [85].

The transfer of Treg cells via TGF-β improves the ConA-induced AIH in mice, but the depletion of Treg cells with anti-CD25 antibodies worsens it [105]. Additionally, interactions between small-molecule mediators and liver-resident cells induce Treg cells in the liver and improve the pathology of ConA-induced hepatitis [60, 106]. In humans, the Treg/Th17 cell balance is decreased, and the number of Treg cells is reduced in the liver [107–109]. Moreover, an inverse correlation between the titers of autoantibodies such as anti-SLA and anti-LKM-1 and the percentage of Treg cells was demonstrated in the liver [107]. Treg cells isolated from patients with AIH exhibit impaired function, including decreased expression of Tim-3, which is involved in their immunosuppressive capacity, and reduced ability to suppress the proliferation and activation of CD4^+^ and CD8^+^ T cells [110]. These findings suggest the role of Treg cells in suppressing AIH pathogenesis in humans as well as mice.

Tregs are decreased in the liver, and their function is impaired in patients with PSC and AIH [111]. In patients with PSC with AIH-associated complications, a reduction in the circulating Treg cell populations and reduced function have been observed, which indicates the involvement of Treg cells in both diseases [107, 110]. Despite an evidently decreased abundance of Treg cells in PBC [112–114], some studies have reported no change or an increase in the number of Treg cells in the blood or the portal region [115, 116]. Comparative analysis of PBC and PSC reflects less pronounced variations and dysfunction of Tregs in PBC and suggests a lower contribution of Tregs to pathogenesis than that in AIH and PSC [114].

Several reports indicate the decreased abundance of Treg cells in the liver in MASH [13, 16, 117]. In mice, the transfer of Treg cells in a MASH model was demonstrated to suppress inflammatory markers such as TNF-α in the liver [13]. Since Th17 cells are important in the pathogenesis of MASH, Treg cells likely control the disease by suppressing Th17 cells, as indicated by several studies evaluating the Th17/Treg balance [118, 119]. However, in humans, the function and role of Tregs in MASH remain partially unclear because another report indicated that the number of liver Tregs is increased in MASLD [120].

In the pathogenesis of fibrosis, Treg cells play a dual role exhibiting anti-inflammatory and fibrosis-promoting effects via TGF-β production. The disease model-based studies reveal that Treg cells promote fibrosis by regulating macrophage phenotype in the CCl4 model while enhancing MMP production by Kupffer cells to promote fibrosis resolution [121]. Furthermore, in the CCl4 model, Treg cells suppress fibrosis-promoting Th2 cells, suggesting their opposing roles in liver fibrosis [122]. Recently, the concept of tissue-resident Tregs (tissue Tregs) expressing the IL-33 receptor ST2 has been reported [123]. Tissue Tregs are present in the mouse liver and their abundance increases in response to IL-33 signaling [123, 124]. Tissue Tregs are important for wound healing. The role of IL-33 signaling in liver fibrosis indicates the potential profibrotic role of tissue Treg [69]. Tregs, including tissue Tregs, contribute to both inflammation and fibrosis, making it challenging to define their role in individual immune-related diseases. Hence, techniques such as spatial transcriptomics to elucidate spatial interactions with other cells in different phases of pathology can further illustrate the interactions.

### Th22 cells

Th22 cells are a subset of CD4^+^ T cells that produce IL-22 and were discovered in 2009 [125]. Th22 cells differentiate from T cells induced by TNF-α and IL-6 signals in an Ahr-dependent manner. Although few reports on the role of Th22 cells in immune-related liver diseases are available, hepatoprotective properties of IL-22 have been reported in mice. Advances in our research focusing on the gut indicate that ILC3 cells is the primary source of IL-22, reflected by their high abundance [126]. This study suggests that *Lactobacillus johnsonii* activates IL-22 production by intestinal ILC3, leading to an increased systemic IL-22 level and suppressed liver inflammation. The mechanism proposed involves the promotion of IL-22-mediated regulatory dendritic cell (DC) accumulation in the liver. Contrastingly, ILC3 comprises a small population in the liver. In the AIH model of ConA-induced hepatitis, CD4 T cells are the primary source of IL-22, and IL-22 deficiency has been reported to exacerbate hepatitis [127]. Additionally, IL-22 overexpression ameliorated ConA-induced hepatitis [128], suggesting the possibility of hepatitis treatment via Th22 induction.

Similarly, IL-22 administration suppresses lipogenesis and steatosis in MASH. Although the ILC3 comprises a small population in the liver, some evidence suggests their importance in the protection against CCl4-induced hepatitis [129]. Thus, it remains undetermined whether Th22 cells or ILC3 are the main players in IL-22-mediated alleviation of various diseases.

Previously, we demonstrated that IL-22 from intestinal ILC3 induced by *Lactobacillus johnsonii* was hepatoprotective [126). Therefore, the biological properties of Th22, IL-22, Th17, and other T cells must be comprehensively investigated in the context of the gut.

### Innate-like T cells (NKT cells, γδ T cells, MAIT cells)

Innate-like T cells are T lymphocytes that constantly express natural killer (NK) cell markers and activated T cell markers and rapidly release large amounts of cytokines when they encounter antigens, playing a role in the innate immune response [130]. These cells are tissue-resident lymphocytes that are abundant in the liver and play an important role in maintaining liver homeostasis, inducing inflammation, and promoting recovery [12, 130]. Similar to Th17 cells, innate-like T cells in the liver effectively produce IL-17, which promotes inflammation and fibrosis.

Invariant NKT cells, a type of innate-like T cell, express a semi-invariant αβTCR and recognize glycolipids presented by the MHC class I molecule CD1d [130, 131]. These cells comprise 30–50% of liver lymphocytes in mice but < 1% of them in humans [132–134]. In AIH models, such as ConA-induced hepatitis [135] and PBC models such as the 2-OA immunization model [136], NKT cells have been identified as important IFN-γ-mediated pathological cells [130]. However, inferring its role in human immune-related liver disease based on the findings in mouse model studies is difficult because its abundance greatly differs between both systems.

MAIT cells, also classified as innate-like T cells, are characterized by the expression of invariant TCR chain [130, 131]. They recognize bacteria-derived ligands from vitamin B2 (riboflavin) or B9 (folate) presented by non-polymorphic MHC Class I (MR1) molecules [137, 138]. In contrast to NKT cells, MAIT cells comprise 10–40% of liver lymphocytes in humans but are almost absent in mice [139, 140]. MAIT cells have been implicated in disease progression via IFN-γ and IL-17 in MASH and via GZMB in AIH and PBC [141, 142]. Furthermore, CCl4-induced liver fibrosis was suppressed in the MR1-blocking condition, suggesting a fibrosis-promoting effect of MAIT cells [143]; however, their contribution to the disease needs to be further clarified. The replication of these human-specific cells in mice is essential for an accurate understanding of the pathogenesis of immune-related liver diseases, which may facilitate future technological advances.

γδ T cells are a subset of T cells with T cell receptors composed of γ and δ chains. They recognize a narrower range of antigens compared to αβ T cells. Like other innate-like T cells, γδ T cells in the liver produce high levels of IL-17 and IFN-γ [130]. However, the anti-fibrotic roles of γδ T cells in immune-related liver diseases were reported in mice. Evaluation of the effects of γδ T cells in the MCD-induced NASH model demonstrated that these cells inhibited the progression of activated stellate cells by inducing apoptosis [144]. Moreover, similar to CD8^+^ Trm cells in the recovery phase, they suppress fibrosis progression through the Fas/FasL signal pathway [144]. Previous studies involving the CCl4 model revealed that γδ T cells can directly kill activated HSCs through NKp46 or other molecules such as FasL and TRAIL [145]. While γδ T cells primarily produce IL-17A, IFN-γ-producing γδ T cells are mainly responsible for killing stellate cells [145].

### Treatment concepts targeting immune-related liver diseases

Unmet medical needs (UMN) and their fulfillment vary across immune-related liver diseases. The need for T cell-targeted therapies largely depends on the availability of other therapies. Targeting the regulation of innate immune cells, such as hepatocytes and macrophages, or the regulation of autoantibody production presents common therapeutic strategies for all these diseases. However, drug development concepts related to T cells have also emerged from research and early stages of development. The unavailability of disease-modifying drugs for MASH implies a long-standing demand for approved drugs that effectively improve liver fibrosis. Recently, GLP-1 agonists to treat underlying obesity and THRβ agonists to inhibit triglyceride accumulation have recently shown remarkable clinical success [146–148]. In addition, peroxisome proliferator-activated receptor (PPAR) agonists that regulate lipid metabolism, inflammation, and fibrosis are under development [148, 149]. Despite the well-known effectiveness of corticosteroids in AIH, unmet needs in terms of treatment options for patients who do not respond to corticosteroids and the management of corticosteroid-induced bone toxicity associated with long-term use should be considered. Several reports demonstrated the high effectiveness of Rituximab in non-responsive patients [150–152]. These reports raise hopes for deep B-cell-targeted therapy in AIH treatment. UDCA is established as the first-line treatment of PBC; however, the establishment of therapeutic agents targeting severe conditions such as fibrosis is needed. No established treatment options modifying the PSC disease state are available; effective treatment is limited to liver transplantation, which is associated with a high rate of post-transplantation recurrence. Therefore, the development of therapeutic agents that can improve the disease state is urgently needed. Currently, Farnesoid X receptor (FXR) agonists are promising for PSC and PBC, whereas PPAR agonists, anti-CCL24 antibodies based on eosinophil control, and apical sodium-dependent bile acid transporter (ASBT) inhibitors that regulate bile acid transporters are under development [153]. However, further research is needed to clarify whether these treatment strategies can fulfill the UMN criteria; moreover, new treatment strategies based on new pathological findings, including T-cell functions, need to be established.

Since Th17 cells are strongly involved in the pathogenesis of PSC, the concept of inhibiting Th17 differentiation with drugs, such as anti-IL-12/23 antibodies, anti-IL-23p19 antibodies, and JAK inhibitors, was initially considered, which are already clinically used for UC. Clinical trials of Ustekinumab in PBC have shown no efficacy [67]; however, the targeting of relevant signals in human immune-related liver diseases has not yet been fully explored. Furthermore, the suppression of liver Th17 cells with RORγt inverse agonists improves the pathophysiology of 3,5-diethoxycarbonyl-1,4-dihydrocollidine (DDC)-induced PSC models transferred with human gut microbiota [14], suggesting that regulation of Th17 differentiation in PSC is promising.

Our group has shown that, in a mouse model of PSC, the administration of bacteriophages that specifically eliminate *Klebsiella pneumonae* (Kp), which is detected at high rates in patients with PSC, improves PSC pathogenesis [154]. Furthermore, inhibiting T cell homing to the liver with α4β7 inhibitors or S1P1 modulators can potentially control the disease. Among the preclinical findings, suppressing hepatic T cell infiltration using anti-α4β7 antibodies in the CCl4 model has been reported to improve the disease state [155]. In PSC, particularly in concurrent UC, it is crucial to standardize concepts that efficiently control the pathophysiology of the gut-liver axis and tissue interactions.

**Fig. 3:**
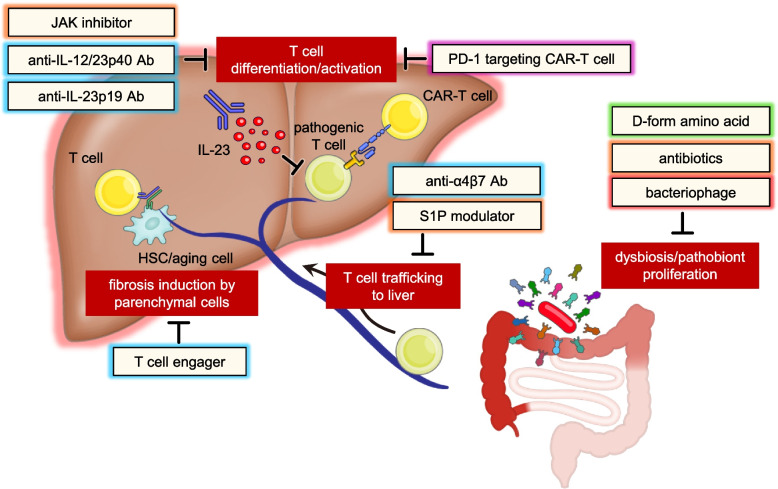
Treatment strategies targeting T cells in immune-related liver diseases An overview of the treatment strategies targeting T cells in immune-related liver diseases. Anti-IL-12/23p40 Ab was tested in clinical trials for PBC but did not exhibit effectiveness [67]. Moreover, the roles of Th17 and Th1 in other immune-related hepatitis conditions like PSC were not clinically validated. JAK inhibitors and anti-IL-23p19 Ab have not been clinically tested; however, similar to the anti-IL-12/23p40 Ab, targeting Th17/Th1 differentiation is considered effective in diseases where it plays a significant role. The effectiveness of PD-1 targeting CAR-T cell therapy has been validated in a PBC mouse model [40], but human study-based validation is pending. Reports on T cell engagers in immune-related hepatitis are currently lacking in both mice and humans. However, based on our research findings showing the importance of the proximity of T cells to HSCs in fibrosis recovery [6], an HSC-T cell engager strategy is anticipated. The effectiveness of anti-α4β7 Ab has been suggested in a mouse model of CCl4-induced fibrosis [155]. Anti-α4β7 Ab and S1P1 modulators, which control T cell homing, have shown efficacy in clinical trials for UC, indicating that they may modify the pathogenesis of PSC. D-form amino acids and bacteriophages are reported to be effective in a mouse PSC model [154, 159], and ongoing clinical trials can validate their efficacy. While the antibiotics has shown effectiveness in patients with PSC [160], their effectiveness in other immune-related hepatitis conditions needs to be confirmed. *CAR* chimeric antigen receptor, *Ab* antibody

Furthermore, the concept of removing activated T cells with anti-PD-1 antibodies has been assessed for autoimmune diseases, such as RA, and these therapies can be expanded to immune-related liver diseases. Recent reports indicated that PD-1 targeting CAR-T cells are effective in eliminating CD8^+^ Trm in PBC models [40]. Moreover, therapy based on uPAR targeting CAR-T cells has been reported to eliminate uPAR-positive senescent cells and improve fibrosis, suggesting the potential for various CAR-T cell applications in the treatment of liver disease [156]. Experience with anti-CD20 antibodies in autoimmune diseases suggests that simple antibodies do not completely remove the target cells from tissues [157]. Therefore, the use of CAR-T cells to eliminate long-term residual cells such as CD8^+^ Trm is crucial for disease control. In recent years, CAR-T cells as well as T cell engagers, which engage and activate T cells near target cells, have been tested in autoimmune diseases, such as SLE, to kill B cells more deeply [158]. Chimeric antigen receptor (CAR) and T cell engagers may be advantageous in immune-related liver diseases, with an increased abundance of T cells in the liver, and the identification of appropriate antigens is desired for this purpose. An overview of the treatment strategies targeting T cells in immune-related liver diseases is shown in Fig. 3.

## Conclusions and future perspectives

In addition to classical T cell subsets, recent reports have proposed human liver-specific and tissue-specific T cell classifications, and the pathogenesis of liver-specific diseases is gradually being clarified. In MASH and PSC, accumulating evidence reflects disease exacerbation due to abnormalities in the cellular immune system, such as CD8^+^ T, Th17, and innate-like T cells. In PBC and AIH, a new mechanism of autoantibody production involving not only Tfh cells but also Tph cells has been implicated. In fibrosis, the involvement of IL-17A from Th17 and innate-like T cells and type II immunity derived from Th2 has been suggested, along with CD8^+^ Trm and γδ T cell-associate anti-fibrotic mechanisms targeting HSCs.

In future analyses, researchers should focus on:

1. Analyses of human tissue samples
2. Unbiased information based on single-cell analyses
3. Integrated spatiotemporal location information, and
4. Collecting comprehensive gene expression data and analyzing crucial cell–cell interactions associated with pathogenesis.Collecting comprehensive gene expression data and analyzing crucial cell–cell interactions associated with pathogenesis.

In the liver, differential functions and roles of macrophages according to the distribution under pathological conditions have been elucidated [3]. Spatial omics analysis of various T cells can potentially elucidate new and different functions and roles of T cells.

## Data Availability

Not applicable.

## Abbreviations

AIH: Autoimmune hepatitis
AMED: Japan Agency for Medical Research and Development
ANCA: Anti-neutrophil cytoplasmic antibodies
ASBT: Apical sodium-dependent bile acid transporter
BCL6: B cell lymphoma 6
BLIMP1: B lymphocyte-induced maturation protein 1
CAR: Chimeric antigen receptor
CDHFD: Choline-deficient high-fat diet
CCl4: Carbon tetrachloride
CXCL: Chemokine (C-X-C motif) ligand
CXCR: Chemokine (C-X-C motif) receptor
DC: Dendritic cell
DDC: 3,5-Diethoxycarbonyl-1,4-dihydrocollidine
FACS: Fluorescence-activated cell sorting
FXR: Farnesoid X receptor
GPR: G protein-coupled receptor
HSC: Hepatic stellate cell
IFN-γ: Interferon gamma
IL: Interleukin
LKM: Liver-kidney microsomal
LSEC: Liver sinusoidal endothelial cell
MAIT: Mucosal-associated invariant T
MASH: Metabolic dysfunction-associated steatohepatitis
MHC: Major histocompatibility complex
MR1: MHC class I-related
NK: Natural killer
NKT: Natural killer T
PC: Plasma cell
PBC: Primary biliary cholangitis
PD-1: Programmed cell death protein 1
PPAR: Peroxisome proliferator-activated receptor
PSC: Primary sclerosing cholangitis
RA: Rheumatoid arthritis
RORγt: Retinoic acid-related orphan receptor gamma t
SCID: Severe combined immunodeficiency
SLA: Soluble liver antigen
STAT: Signal transducer and activator of transcription
TAA: Thioacetamide
Tfh: Follicular helper T
TIGIT: T cell immunoreceptor with Ig and ITIM domains
TLO: Tertiary lymphoid organ
Tph: Peripheral helper T
Trm: Tissue resident memory
TSLP: Thymic stromal lymphopoietin
UDCA: Ursodeoxycholic acid
